# Regional level risk factors associated with the occurrence of African swine fever in West and East Africa

**DOI:** 10.1186/s13071-016-1953-z

**Published:** 2017-01-07

**Authors:** Zheng Y. X. Huang, Frank van Langevelde, Karanina J. Honer, Marc Naguib, Willem F. de Boer

**Affiliations:** 1 College of Life Sciences, Nanjing Normal University, 210023 Nanjing, China; 2 Resource Ecology Group, Wageningen University, 6708PB Wageningen, The Netherlands; 3 Behavioural Ecology, Wageningen University, 6708WD Wageningen, The Netherlands

**Keywords:** Domestic cycle, Sylvatic cycle, Wild suid, Giant forest hog, Habitat fragmentation, *Ornithodoros moubata*

## Abstract

**Background:**

African swine fever (ASF) causes severe socio-economic impacts due to high mortality and trade restrictions. Many risk factors of ASF have been identified at farm level. However, understanding the risk factors, especially wild suid hosts, determining ASF transmission at regional level remains limited.

**Methods:**

Based on ASF outbreak data in domestic pigs during 2006–2014, we here tested, separately for West and East Africa, which risk factors were linked to ASF presence at a regional level, using generalized linear mixed models.

**Results:**

Our results show that ASF infections in the preceding year was an important predictor for ASF presence in both West and East Africa. Both pig density and human density were positively associated with ASF presence in West Africa. In East Africa, ASF outbreaks in domestic pigs were also correlated with higher percentages of areas occupied by giant forest hogs and by high-tick-risk areas.

**Conclusions:**

Our results suggest that regional ASF risk in East Africa and in West Africa were associated with different sets of risk factors. Regional ASF risk in West Africa mainly followed the domestic cycle, whereas the sylvatic cycle may influence regional ASF risk in East Africa. With these findings, we contribute to the better understanding of the risk factors of ASF occurrence at regional scales that may aid the implementation of effective control measures.

**Electronic supplementary material:**

The online version of this article (doi:10.1186/s13071-016-1953-z) contains supplementary material, which is available to authorized users.

## Background

African swine fever (ASF), caused by a DNA virus of the genus *Asfivirus*, is a highly contagious disease for domestic pigs. It can have severe socio-economic impacts due to high mortality and trade restrictions [1]. ASF is considered the major limiting factor for pig production in many sub-Saharan African countries where a significant commercial pig industry exists [1, 2]. ASF has also spread outside Africa, including recently to the Caucasus and Russia, posing considerable threats to the global pig industry [3–6]. Because no vaccine has been developed, a full understanding of the ecology and epidemiology of ASF is fundamental to implement effective control measures [2, 6, 7].

ASF outbreaks have been reported for many sub-Saharan African countries, and the epidemiology and ecology of ASF are thought to vary between different regions [8, 9]. Generally, it has been considered that ASF virus can circulate through two cycles in sub-Saharan Africa, the sylvatic cycle and the domestic cycle [2, 9, 10]. The sylvatic cycle has been documented in some Eastern and Southern African countries [7, 9–12]. It involves wild suids as the host, including the common warthog (*Phacochoerus africanus*), and is spread *via* soft ticks (*Ornithodoros moubata*) to domestic pigs making the virus persistent [13]. However, in the domestic cycle ASF virus mainly spreads *via* direct contact between domestic pigs or between pork products and pigs. A recent study assessed the suitability of these two transmission pathways in Africa and concluded that the domestic cycle may occur throughout sub-Saharan Africa, whereas the sylvatic cycle might be found mainly in East, Central and Southern Africa, where warthogs are present [8].

A few studies have been conducted to investigate the factors linked with ASF risk [10]. For the domestic cycle, the density or the herd size of domestic pigs has been considered to be positively associated with ASF outbreaks at farm level [9, 14]. Farms with traditional free-ranging husbandry systems typically experience higher ASF risk, presumably due to low biosecurity [15]. Previous occurrence of the disease on a farm and infected neighbouring farms has also been found to promote the chance of ASF infection [16]. In addition to these risk factors, ASF spread in the domestic cycle has been linked to trade-related factors [16, 17], such as the density of the road network and water bodies [14]. In fact, the movements of infected domestic pigs might be the most important factor for ASF spread in some regions, especially in West Africa where the domestic cycle is considered the only transmission pathway [7, 10, 18]. For the sylvatic cycle, the common warthog has been considered the most important vertebrate host for the circulation of ASF virus [13, 19]. Interspecific transmission between warthogs and domestic pigs may occur indirectly through *Ornithodoros* ticks, especially when pigs that share grazing areas with warthogs are bitten by infected ticks, or during human interference when warthog carcasses with infected ticks are transported [10]. Bushpigs (*Potamochoerus larvatus*) can also spread ASF to domestic pigs *via* ticks, but they are generally assumed less important than warthogs due to their lower population densities, nocturnal habits, and limited contact with pigs and soft ticks [13, 20]. Finally, the red river hog (*Potamochoerus porcus*) and the giant forest hog (*Hylochoerus meinertzhangeni*) can also be infected [13]. However, neither species seems to play an important role in ASF transmission due to their limited/restricted distributions [13].

To date, risk factors have predominantly been identified at farm level, whereas the influence of risk factors such as the role of wild suids and tick distribution on the dynamics of ASF transmission at larger spatial scales is not well understood [8, 10]. Understanding regional risk factors of infectious diseases is necessary for revealing the underlying epidemiological processes that might lead to important implications for control at regional scales [1, 21, 22]. Therefore, the present study aims to identify the risk factors that are associated with ASF occurrence at a regional scale in Africa. Due to the potentially different ASF transmission pathways, we compare the risk factors between West Africa and East Africa. We expect that the presence of wild suids and tick distribution is positively associated with the probability of ASF occurrence in East Africa, whereas domestic pig density and indicators of trade activities have a positive effect in West Africa.

## Methods

### African swine fever data

Data on ASF presence/absence in domestic pigs for some countries in the sub-Saharan Africa from 2005 to 2014 is available in the World Animal Health Information (http://www.oie.int/wahis_2/public/wahid.php/Wahidhome/Home) from the World Organization for Animal Health (OIE) [23], which provides data on ASF outbreaks at a temporal resolution of 6 months. Some African countries reported ASF outbreaks only at country level, while other countries specified ASF outbreaks at a smaller administrative level. The smallest administrative level of reporting was used as the level of analyses in this study, whilst those countries reporting ASF outbreaks only at country level were excluded from the study. Only the countries with “ASF history” (at least one outbreak was reported during the study period) were included in the analyses, as this excluded those countries which had possibly ASF outbreaks but never reported them, so that we avoided some false absence data. Thus, we assumed that no report from a country/administrative unit that had previously reported ASF is indicative of the absence of disease for that year. In addition, to determine the effect of previous infection status, we only involved the administrative areas from the countries that reporting ASF outbreaks in more than two consecutive years. Areas without domestic pigs were excluded. The data set yielded from OIE included eleven countries (Angola, Benin, Burkina Faso, Cameroon, Ghana, Mozambique, Malawi, Nigeria, Rwanda, Togo, and Zambia). As we compared East Africa and West Africa, we then, according to the definition of Statistics Division of the United Nations (http://millenniumindicators.un.org/unsd/methods/m49/m49.htm), excluded Angola and divided the remaining ten countries into East Africa and West Africa. The final dataset (Additional file 1: Table S1, Figure S1) for the analyses of West Africa consisted of 1287 cases of ASF presence/absence data covering six countries and 177 administration areas, and the final dataset for East Africa included 394 cases of presence/absence data covering four countries and 51 administration areas.

### Data of the predictors

As the infection status of the previous year (PreInf) has been linked to ASF risk in earlier studies, we tested its effect to determine the dependency of ASF status between years. We also tested for the effect of the domestic pig density (Pig) on the risk of ASF occurrence. As indicators of trade activities, road density (Road) and human population density (Human) were calculated for each administrative area (Table 1).

**Table 1:** Description and summary (mean ± standard deviation, SD) of predictors used in the analyses for West and East Africa, with abbreviation and unit

Description of data	Abbreviation	Units	Mean ± SD	
			West Africa	East Africa
*Biotic variables*				
Pig density	Pig	*n*/km^2^	561 ± 732	885 ± 1679
Common warthog	WarthogC	–	0.47 ± 0.46	0.86 ± 0.15
Bushpig	BushpigC	–	na^a^	0.91 ± 0.12
Red river hog	RiverhogC	–	0.45 ± 0.47	na^a^
Giant forest hog	ForesthogC	–	0.06 ± 0.21	0.31 ± 0.41
Human population density	Human	*n*/km^2^	178 ± 286	273 ± 310
Road density	Road	km/km^2^	15.7 ± 14.5	64.6 ± 65.8
Mean tick risk	TickRiskMean	–	na^a^	0.26 ± 0.12
Area with high-tick-risk	TickRisk2	–	na^a^	0.44 ± 0.35
Area with high-tick-risk	TickRisk4	–	na^a^	0.17 ± 0.20
Area with high-tick-risk	TickRisk6	–	na^a^	0.03 ± 0.10
*Habitat related variables*				
Percentage of habitat area	Habitat	–	0.87 ± 0.21	0.73 ± 0.20
Measure of the degree of isolation and fragmentation	MPI	–	886 ± 2563	4214 ± 7233
Mean nearest neighbour	MNN	m	750 ± 1041	1611 ± 1036
Percentage of protected area	PDA	–	0.18 ± 0.23	0.17 ± 0.16
*Climatic variables*				
Annual mean temperature	TemMean	°C	28.2 ± 1.08	20.9 ± 2.17
Mean temperature in preceding year	PreTemMean	°C	27.9 ± 1.09	21.1 ± 2.13
Annual mean precipitation	RainMean	mm	101 ± 31.1	101 ± 24.3
Mean precipitation in preceding year	PreRainMean	mm	97.4 ± 32.0	94.0 ± 22.0

^a^ There is no bush pig in West Africa and no red river hog in East Africa, tick risk variables were not included in the analyses of West Africa

Since ASF risk might be influenced by the occurrence of some wild suid species, we calculated the percentage in each administrative area that is occupied by the common warthog, the bushpig, the red river hog, and the giant forest hog and tested whether or not the wild suid species occurrences were correlated with ASF occurrence. The distributions of these species were obtained from the African Mammal Databank (AMD), a GIS-based databank of the distribution of medium to large mammals in Africa [24, 25]. For each species, AMD includes two polygon coverage files respectively describing the distribution of suitable habitat and the distribution of species occurrence at a 1 × 1 km resolution [24, 25]. The intersection of these two distribution maps was calculated as the ‘actual distribution’ for each species (Boitani et al. [25]). The percentage of protected area within an administrative area (PDA) was also included in the analysis since it was expected to hold a higher density of wild suids, increasing ASF risk. Habitat structure can influence ASF transmission by affecting host distribution and movements [13, 26]. Therefore, some habitat related predictors were incorporated in the analyses. For each administrative area, we first calculated the percentage of suitable habitat (Habitat) based on a detailed (300 m) global land cover dataset (GlobCover by European Space Agency). Twelve out of 23 land-use types were combined to calculate the areas that can sustain grazers (see Additional file 1: Table S2), i.e. grassland and open woodland that are used by livestock and several of the most important wild hosts. Based on the map of grazing area, we calculated the Mean Nearest Neighbour (MNN) and Mean Proximity Index (MPI) to test the effect of fragmentation and isolation on the risk of ASF occurrence. MNN, as a measure of patch isolation, represents the average shortest distance of patches that can sustain grazers in an administrative area to the closest similar patch. For each patch, the size and mean distance to all neighbouring patches of the same type was calculated to provide the index of MPI, which measures the degree of isolation and fragmentation [27].

In addition, climate can affect ASF transmission dynamics by influencing the biology of the host, pathogen, and vector [28, 29]. Precipitation and temperature can affect ASF transmission by influencing the movements and habitat use of wild suid species. For example, in the dry season and under high temperature conditions, wild suids aggregate at small water ponds, which might facilitation ASF transmission [30]. Therefore, the annual mean temperature and mean precipitation (Table 1) were calculated from the Climate Research Unit (CRU) datasets [31], a time-series dataset that yields month-by-month variations in climate from 1900 to 2010 with a grid cell of 0.5 × 0.5 degree. Because of the adaptive process of hosts and ticks, the climatic variables might show lag-effects on pathogen transmission [32, 33]. We thus also tested the effects of the temperature and precipitation conditions in the preceding year.

### Map of tick risk

To investigate the role of the sylvatic cycle, we also tested the effect of the distribution of the tick vector *O. moubata* on ASF spread at the regional scale. *O. moubata* is considered to be an important tick vector for ASF [13]. The presence data of *O. moubata* (more than 200 records) was accessed through the VectorMap data portal (http://www.vectormap.org). Then we applied species distribution modelling with a machine learning technique, namely maximum entropy modeling (Maxent v.3.3.3 k) [34] to generate the distribution map of *O. moubata,* using 19 data layers as predictors (Additional file 1: Table S3) containing bioclimatic variables at 30 arc-seconds spatial resolution, acquired from the WorldClim dataset (http://worldclim.org). The model with 10 subsampling runs using a random test percentage of 20% was constructed with default settings in Maxent [35]. We included all variables as a full model and did not remove highly correlated predictors, because we focused on the explanatory power of the model and multicollinearity is known to have little effect on model fit [36]. The average AUC score (the area under the Receiver Operating Characteristic curve) for the full model was 0.928 ± 0.010. Based on this model, we generated a map representing the probabilities of tick presence (Additional file 2: Figure S1).

With the tick distribution map, we averaged the probabilities of tick presence for each administrative area (TickRiskMean). We also arbitrarily set three probability values (0.2, 0.4 and 0.6) as the thresholds of high-tick-risk, and consequently calculated the percentages of area with high-tick-risk (areas with the the probability of tick presence higher than 0.2, 0.4, and 0.6, respectively) for each administrative area, and got another three measures of tick risk (TickRisk2, TickRisk4, TickRisk6). As *O. moubata* was predicted to be present in very few administrative areas in West Africa (Additional file 2: Figure S1), the tick risk variables were only included in the analyses of East Africa. In addition, to test the effect of tick-suid interactions, we also included the interaction terms between tick risk variables and wild suid variables.

The data for all predictor variables were acquired or generated from existing databases (Additional file 1: Table S4). All data pre-processing was carried out in ArcGIS v10.0.

### Statistical analyses

Generalized linear mixed models (GLMM) with a binary response (logit link) were used to examine the effects of predictors on the probability of ASF occurrence. Country was included in the models as a random factor to control for possible differences between countries, thereby correcting for the effect of differences in veterinary service efficiency and used control measures. Only the ASF presence/absence data from 2006 to 2014 were used as the dependent variable, because the earliest year in the dataset was 2005, which was used to calculate the previous infection status (PreInf) for 2006. Before performing the GLMMs, we log-transformed Pig, Human, Road, MPI and MNN, making these variables closer to normal distribution. All continuous variables were rescaled to have a mean of zero and a standard deviation of one.

Using GLMMs, we first performed univariate analyses to identify the potential risk factors. The area of the unit (Area) was retained in the model as an obligate variable to correct for the effect of area size. Variables with a *P*-value of less than 0.15 were identified as potential risk factors, which were used to construct multiple regression models. We then assessed multi-collinearity by examining the variance inflation factor (VIF) of the candidate variables. For highly correlated independent variables, only the one with the smallest *P*-value in the univariate analyses was maintained in fitting the multiple regression models to avoid multi-collinearity. To construct the final multiple model, we used both backward and forward selection, where the likelihood ratio test was applied to test for difference in the fit of the nested models. For both West and East Africa, backward and forward selection generated same models. We then included interaction terms after including all main factors. Main terms were maintained in the model if they were included in a significant interaction term. We tested for the spatial autocorrelation of the residuals (of the final multiple model) using Moran’s *I* index and found little evidence of spatial autocorrelation (Additional file 2: Table S1). The AUC scores for the final models were also reported to assess the goodness-of-fit. The whole statistical processes were conducted in R 2.15.1 with *lme4* [37].

## Results

### Descriptive epidemiology

Over the entire study period, 25.0% of the administrative areas in West Africa reported ASF occurrence, whereas in East Africa this was 21.0% (Table 2).

**Table 2:** The number of infected areas, regional prevalence of ASF occurrence in West and East Africa

	West Africa			East Africa		
Year	No. of area	Infected areas	Infection prevalence (%)	No. of area	Infected areas	Infection prevalence (%)
2005	13	7	0.538	98	25	0.255
2006	51	12	0.235	98	16	0.163
2007	51	9	0.176	177	34	0.192
2008	51	11	0.216	177	35	0.198
2009	51	9	0.176	177	40	0.226
2010	51	18	0.353	177	52	0.294
2011	44	12	0.273	116	17	0.147
2012	44	9	0.205	151	31	0.205
2013	44	18	0.409	151	35	0.232
2014	44	6	0.136	151	25	0.166
Total	444	111	0.250	904	202	0.210

### Univariate analyses of risk factors

Twenty potential risk factors were individually tested using univariate regression analyses (Table 3). Previous infection status (PreInf) was positively associated with ASF occurrence in domestic pigs both in West and East Africa. Besides, five biotic factors (Pig, Human, Road, WarthogC, and RiverhogC), four climatic factors and one related to grazing area (Habitat) were significantly correlated with ASF occurrence in West Africa. In East Africa, besides PreInf, only three biotic variables (ForesthogC, WarthogC and TickRisk4) and two climatic variables (TemMean and PreTemMean) were significantly associated with ASF occurrence in domestic pigs.

**Table 3:** Summary statistics (standardized regression coefficient *b*, *Z*-value and *P*-value) for the potential predictors correlated with the occurrence of African swine fever in univariate analyses for West and East Africa. For explanation of the variables, see Table 2

	West Africa (*n* = 727)			East Africa (*n* = 217)		
Variables	*b*	*Z*-value	*P*-value	*b*	*Z*-value	*P*-value
PreInf	1.64	10.03	< 0.001***	1.02	3.88	< 0.001***
Pig	0.404	3.97	< 0.001***	-0.026	-0.15	0.880
WarthogC	-0.27	-3.62	0.003**	-0.24	-2.03	0.043*
BushpigC	na^a^	na^a^	na^a^	0.026	0.189	0.850
RiverhogC	0.363	3.77	0.001*	na^a^	na^a^	na^a^
ForesthogC	0.128	1.453	0.146	0.81	3.04	0.002**
TickRiskMean	na^a^	na^a^	na^a^	2.44	1.47	0.14
TickRisk2	na^a^	na^a^	na^a^	0.32	0.54	0.59
TickRisk4	na^a^	na^a^	na^a^	1.99	2.38	0.017*
TickRisk6	na^a^	na^a^	na^a^	2.33	1.61	0.11
Human	0.384	4.71	< 0.001***	0.42	1.37	0.170
Road	0.530	4.34	< 0.001***	0.522	1.12	0.264
Habitat	-0.304	-4.00	< 0.001**	0.040	0.293	0.769
MPI	0.044	0.500	0.616	0.311	1.11	0.267
MNN	0.116	1.33	0.184	0.240	1.32	0.186
PDA	-0.089	-1.08	0.280	0.308	1.93	0.053
TemMean	-0.21	-2.01	0.043*	0.213	0.608	0.543
PreTemMean	-0.29	-2.75	0.006**	0.268	0.737	0.461
RainMean	0.34	3.61	0.001***	0.052	0.346	0.729
PreRainMean	0.19	2.12	0.033*	0.112	0.720	0.472

^a^There is no bush pig in West Africa and no red river hog in East Africa. Tick risk was not included in the analyses in West Africa

*P < 0.05; **P < 0.01; ***P < 0.001

### Multiple regression models

After checking for collinearity, nine variables (PreInf, Pig, WarthogC, RiverhogC, Human, Road, Habitat, PDA, PreTemMean and RainMean) were retained to construct the final multiple regression models for West Africa, and five (PreInf, WarthogC, ForesthogC, TickRisk4, and PDA) were retained for East Africa. For West Africa, the results of stepwise model selection (Table 4) showed that ASF occurrence in domestic pigs was positively associated with previous infection status (PreInf, OR = 4.63, 95% CI: 3.92–5.46, *P* < 0.001), pig density (Pig, OR = 1.32, 95% CI: 1.19–1.46, *P* = 0.007) and human population density (Human, OR = 1.36, 95% CI: 1.23–1.51, *P* = 0.002), though many variables were significantly correlated with ASF presence in the univariate analysis. The area under the ROC curve (AUC) for the final multiple model for West Africa was 0.785. For East Africa, previous infection (PreInf, OR = 2.40, 95% CI: 1.84–3.14, *P* = 0.001), percentage of the area occupied by forest hog (ForesthogC, OR = 2.14, 95% CI: 1.63–2.81, *P* = 0.005), and the percentage of area with high-tick-risk (TickRisk4, OR = 6.98, 95% CI: 1.22–39.85, *P* = 0.029) had positive relationships with ASF occurrence in domestic pigs. No interaction terms was significantly correlated with ASF presence in East Africa. The AUC for the final multiple model in East Africa was 0.780.

**Table 4:** Summary statistics (standardized regression coefficient *b* ± standard error, SE; Odds Ratio, OR, 95% CI, and *P*-value) of the model averaging analyses on the occurrence of African swine fever in West and East Africa. Variables are previous infection status (PreInf), pig density (Pig), human population density (Human), the percentage of the area occupied by giant forest hog (ForesthogC), and the percentage of area with high-tick-risk (TickRisk4)

	West Africa (*n* = 727)			East Africa (*n* = 217)		
Variables	*b* ± SE	OR (95% CI)	*P*-value	*b* ± SE	OR (95% CI)	*P*-value
PreInf	1.53 ± 0.16	4.63 (3.92–5.46)	< 0.001***	0.88 ± 0.27	2.40 (1.84–3.14)	0.001**
Pig	0.28 ± 0.10	1.32 (1.19–1.46)	0.007**			
Human	0.31 ± 0.10	1.36 (1.23–1.51)	0.002**			
ForesthogC				0.76 ± 0.27	2.14 (1.63–2.81)	0.005**
TickRisk4				1.94 ± 0.89	6.98 (1.22–39.85)	0.029*

*P < 0.05; **P < 0.01; ***P < 0.001

## Discussion

Many previous studies have been conducted to investigate risk factors for ASF transmission at farm level [9, 10, 14, 16]. However, understanding of the factors affecting regional disease risk remains limited. Here, we tested which factors were correlated with the probability of ASF presence in domestic pigs at regional scale in Africa. Our study identified different sets of risk factors in West Africa and East Africa. Previous infection status was positively correlated with ASF occurrence in domestic pigs in both West and East Africa, suggesting that for both West and East Africa greater effort should be made to control ASF in those areas that have experienced ASF outbreaks in the past. Pig density and human density were also positively associated with regional ASF occurrence in West Africa, where ASF transmission follows the domestic cycle. In East Africa, the percentage of the area where giant forest hog occurred, and the high-tick-risk area, were positively linked to ASF occurrence in domestic pigs, suggesting that the sylvatic cycle is of epidemiologic importance here.

### West Africa

In line with previous studies [10, 16], the effect of the previous infection status (PreInf) on ASF presence in domestic pigs in West Africa indicates that ASF occurs repeatedly in the same area. In West Africa, where the domestic cycle dominates, the endemicity of ASF might be caused by the long persistence of the ASF virus which, shed by infected pigs or asymptomatic carriers, can remain in the environment for a long time [38]. In addition, the significant relationship of previous infection status might be caused by those factors that were not considered in the analyses but associated with the previous ASF occurrence, such as within-country variation in the quality of veterinary service or used control measures.

We showed that both pig density and human density were positively correlated with regional ASF occurrence in West Africa, which was also in line with previous studies [9], suggesting that the domestic cycle is indeed dominant in West Africa. A higher pig density implies a higher probability for susceptible pigs to contact infected pigs or pork products [39], contributing to the spread and persistence of ASF virus [9, 40]. Thus, the probability of ASF presence is higher with increasing pig density. Higher human population density, which was used as the surrogate of trade activity [41, 42], may also facilitate ASF spread. In fact, pig movement and trade activity have been considered as important factors for ASF spread, especially in West Africa where the domestic cycle is dominated [10, 11, 17]. As expected, no wild suids showed a significant relationship with ASF occurrence, suggesting that the sylvatic cycle may not play an important role in ASF transmission at regional scales in West Africa.

### East Africa

Our results from East Africa also identified previous infection status (PreInf) as a predictor for ASF occurrence in domestic pigs at regional scales. The endemicity of ASF in East Africa might be stimulated by the sylvatic cycle where infected wild suids and ticks can maintain the virus, transmit the virus to domestic pigs, and contribute to ASF persistence. Asymptomatic carriers of wild suids may also facilitate the persistence of ASF in East Africa [10]. We also found a positive relationship between ASF risk and the area with high risk of ticks, indicating that the sylvatic cycle may play an importance role in ASF spread in East Africa. Previous studies have documented the importance of warthogs in the sylvatic cycle in East Africa. However, we did not find a significant effect of warthog distribution in East Africa. This might be caused by the limitation of our warthog data. Due to the lack of the data of warthog densities, we could only use the percentage of area covered by warthog distribution (WarthogC) as a surrogate to test for the effect of warthog on ASF occurrence. However, in our analyses all administrative areas in East Africa had an occurrence of warthog (WarthogC > 0). The small variation of WarthogC (86 ± 15% SD, Table 1) precluded it to be a good variable to test for the effect of warthogs.

It has been considered that the giant forest hog is unlikely to play an important role in ASF transmission as its distribution is restricted to areas of dense forest where domestic pig production is not common [13]. However, here we show a significant positive relationship between ASF presence and the occurrence of giant forest hog (ForesthogC), indicating that giant forest hog might promote ASF risk at a regional scale in East Africa. This suggests the presence of the sylvatic cycle in East Africa. At a regional scale, giant forest hog may promote an increase in the density of tick vectors or transmit ASF to other wild suid species that share the same habitats, and thus increases regional ASF risk. Certainly, the positive effect of giant forest hog might be caused by some unidentified factors that was correlated with giant forest hog. For example, it could be that in areas where forest hog occur there are high densities of warthog that facilitate ASF transmission. In any case, more study is needed to better understand the effect of giant forest hog on ASF transmission at regional scale.

Conclusions are not easy to be drawn from large-spatial-scale, correlative studies with data from different sources, due to the complexity of the natural environment and the difficulty of controlling confounding factors [43]. Some variables, such as the quality of the veterinary services or used control measures, could not be taken into account because of lack or incompleteness of data. However, the random factor country used in the analyses, controlled, to some extent, for the variation caused by these variables at country level. In addition, the difficulties of getting harmonized and accurate data of ASF presence/absence might influence the precision of this analysis. Especially, the information from the OIE system might under-estimated the true ASF outbreaks because of several reasons, such as clandestine pig sales or ineffective reporting systems due to lack of budget or change in administration. This under-reporting issue can limit the generality of our results. Despite these limitations, our study, for the first time, tests for the effect of biotic and climatic factors on ASF presence at regional level in Africa.

## Conclusions

Our study showed that the factors that play an important role in ASF transmission at farm level, like previous infection status and domestic pig density, also link to ASF disease dynamics at regional level. We also demonstrated that ASF occurrence in East and West Africa was associated with different sets of predictors. For West Africa, we found support for the importance of the domestic cycle in ASF transmission where the ASF virus mainly spreads via direct contact between domestic pigs or between pork products and pigs. In East Africa, we show that the distribution of the tick vector and the giant forest hog were correlated to the probability of ASF presence at regional level, but more efforts are still needed to better understand the sylvatic cycle and the role of wild suids in the epidemiology of ASF at this level. Our results are relevant for developing more effective control strategies for ASF spread as they highlight variation in the regional correlates of ASF outbreaks.

## Additional files

Additional file 1: Additional information for data used in analyses. (DOCX 242 kb)
Additional file 2: Additional information for the results of analyses. (DOCX 154 kb)

## Abbreviations

AMD: African mammal databank
ASF: African swine fever
AUC: Area under ROC curve
CI: Confidence interval
CRU: Climate Research Unit
GLMM: Generalized linear mixed model
OIE: World Organization for Animal Health
OR: Odds ratio
ROC curve: Receiver operating characteristic curve
SD: Standard deviation
VIF: Variance inflation factor
