# Supplementary material for: Regional level risk factors associated with the occurrence of African swine fever in West and East Africa
Source: Parasit Vectors. 2017 Jan 7;10:16. doi: 10.1186/s13071-016-1953-z (PMC5219763; doi:10.1186/s13071-016-1953-z)

# Supplementary Information (Additional File 1)

# Regional level risk factors associated with the occurrence of African swine fever in West and East Africa

Zheng Y.X. Huang^1,2*^, Frank van Langevelde^2^, Karanina J. Honer^2,3^, Marc Naguib^3^and Willem F. de Boer^2^

^1^College of Life Sciences, Nanjing Normal University, 210023 Nanjing, China

^2^Resource Ecology Group, Wageningen University, 6708PB Wageningen, the Netherlands

^3^Behavioural Ecology, Wageningen University, 6708WD Wageningen, the Netherlands

*Correspondence: zhengyxhuang@gmail.com

**Table S1:** The names of the countries (with their country codes and the number of administrative areas) and years of the ASF presence data used in the analyses

| **West/East Africa** | **Country name** | **Country code** | **Years** | **Number of areas** |
| --- | --- | --- | --- | --- |
| West | Benin | BEN | 2005-2014 | 73 |
| West | Burkina Faso | BFA | 2007-2014 | 44 |
| West | Cameroon | CMR | 2005-2014 | 10 |
| West | Ghana | GHA | 2005-2014 | 10 |
| West | Nigeria | NGA | 2007-2014 | 35 |
| West | Togo | TGO | 2006-2014 | 5 |
| East | Malawi | MWI | 2005-2014 | 3 |
| East | Mozambique | MOZ | 2005-2014 | 10 |
| East | Rwanda | RWA | 2006-2014 | 29 |
| East | Zambia | ZMB | 2006-2014 | 9 |

**Table S2:** All land use/cover types from the global land cover dataset (European Space Agency) and the suitable types used for the habitat of wild hosts.

| **Land use/cover type** | **Habitat** |
| --- | --- |
| Post-flooding or irrigated croplands (or aquatic) | Y |
| Rainfed croplands | Y |
| Mosaic cropland (50-70%) / vegetation (grassland/shrubland/forest) (20-50%) | Y |
| Mosaic vegetation (grassland/shrubland/forest) (50-70%) / cropland (20-50%) | Y |
| Closed to open (>15%) broadleaved evergreen or semi-deciduous forest (>5m) |  |
| Closed (>40%) broadleaved deciduous forest (>5m) |  |
| Open (15-40%) broadleaved deciduous forest/woodland (>5m) | Y |
| Open (15-40%) needleleaved deciduous or evergreen forest (>5m) | Y |
| Closed to open (>15%) mixed broadleaved and needleleaved forest (>5m) | Y |
| Mosaic forest or shrubland (50-70%) / grassland (20-50%) | Y |
| Mosaic grassland (50-70%) / forest or shrubland (20-50%) | Y |
| Closed to open (>15%) (broadleaved or needleleaved, evergreen or deciduous) shrubland (<5m) | Y |
| Closed to open (>15%) herbaceous vegetation (grassland, savannas or lichens/mosses) | Y |
| Sparse (<15%) vegetation |  |
| Closed to open (>15%) broadleaved forest regularly flooded (semi-permanently or temporarily) - Fresh or brackish water |  |
| Closed (>40%) broadleaved forest or shrubland permanently flooded - Saline or brackish water |  |
| Closed to open (>15%) grassland or woody vegetation on regularly flooded or waterlogged soil - Fresh, brackish or saline water | Y |
| Artificial surfaces and associated areas (Urban areas >50%) |  |
| Bare areas |  |
| Water bodies |  |
| No data (burnt areas, clouds,…) |  |

**Table S3:** 19 bioclimatic variables used in modelling tick distribution in Africa

| Layers | Description |
| --- | --- |
| BIO1 | Annual Mean Temperature |
| BIO2 | Mean Diurnal Range (Mean of monthly (max temp - min temp)) |
| BIO3 | Isothermality (BIO2/BIO7) (* 100) |
| BIO4 | Temperature Seasonality (standard deviation *100) |
| BIO5 | Max Temperature of Warmest Month |
| BIO6 | Min Temperature of Coldest Month |
| BIO7 | Temperature Annual Range (BIO5-BIO6) |
| BIO8 | Mean Temperature of Wettest Quarter |
| BIO9 | Mean Temperature of Driest Quarter |
| BIO10 | Mean Temperature of Warmest Quarter |
| BIO11 | Mean Temperature of Coldest Quarter |
| BIO12 | Annual Precipitation |
| BIO13 | Precipitation of Wettest Month |
| BIO14 | Precipitation of Driest Month |
| BIO15 | Precipitation Seasonality (Coefficient of Variation) |
| BIO16 | Precipitation of Wettest Quarter |
| BIO17 | Precipitation of Driest Quarter |
| BIO18 | Precipitation of Warmest Quarter |
| BIO19 | Precipitation of Coldest Quarter |

**Table S4:** Formats, abbreviations and sources of the original data for the variables we used in the analyses

| Datebase | Format | Variables | Data source | Web site links |
| --- | --- | --- | --- | --- |
| ASF outbreaks | binary | PreInf | OIE | http://www.oie.int/wahis_2/public/wahid.php/Diseaseinformation/statusdetail |
| Pig density | raster | Pig | FAO | http://harvestchoice.org/data/an05_pig |
| Human density | raster | Human | NASA | http://sedac.ciesin.columbia.edu/data/set/gpw-v3-population-density |
| Protected area | polygon | PDA | Protected Planet | http://www.protectedplanet.net/ |
| Road density | raster | Road | OpenStreetMap | http://www.openstreetmap.org/#map=10/-23.9034/35.1604 |
| Land cover dataset | raster | Habitat  MNN  MPI | GlobCover by  ESA | http://www.esa.int/Our_Activities/Observing_the_Earth/Space_for_our_climate/ESA_global_land_cover_map_available_online |
| Wild suid distribution | polygon | WarthogC  ForesthogC  RiverhogC  BushpigC | AMD | http://www.gisbau.uniroma1.it/amd/homepage.html |
| Time-series of climatic data | raster | TemMean,  RainMean,  PreTemMean,  PreRainMean | CRU | http://badc.nerc.ac.uk/view/badc.nerc.ac.uk__ATOM__dataent_1256223773328276 |
| Tick risk | dataset | TickRiskMean  TickRisk3  TickRisk4  TickRisk7 | vector map | http://vectormap.si.edu/index.htm |

**Figure S1:** Study areas (including A: West Africa and B: East Africa) for the analyses. Grey curves are the borders of administrative areas.


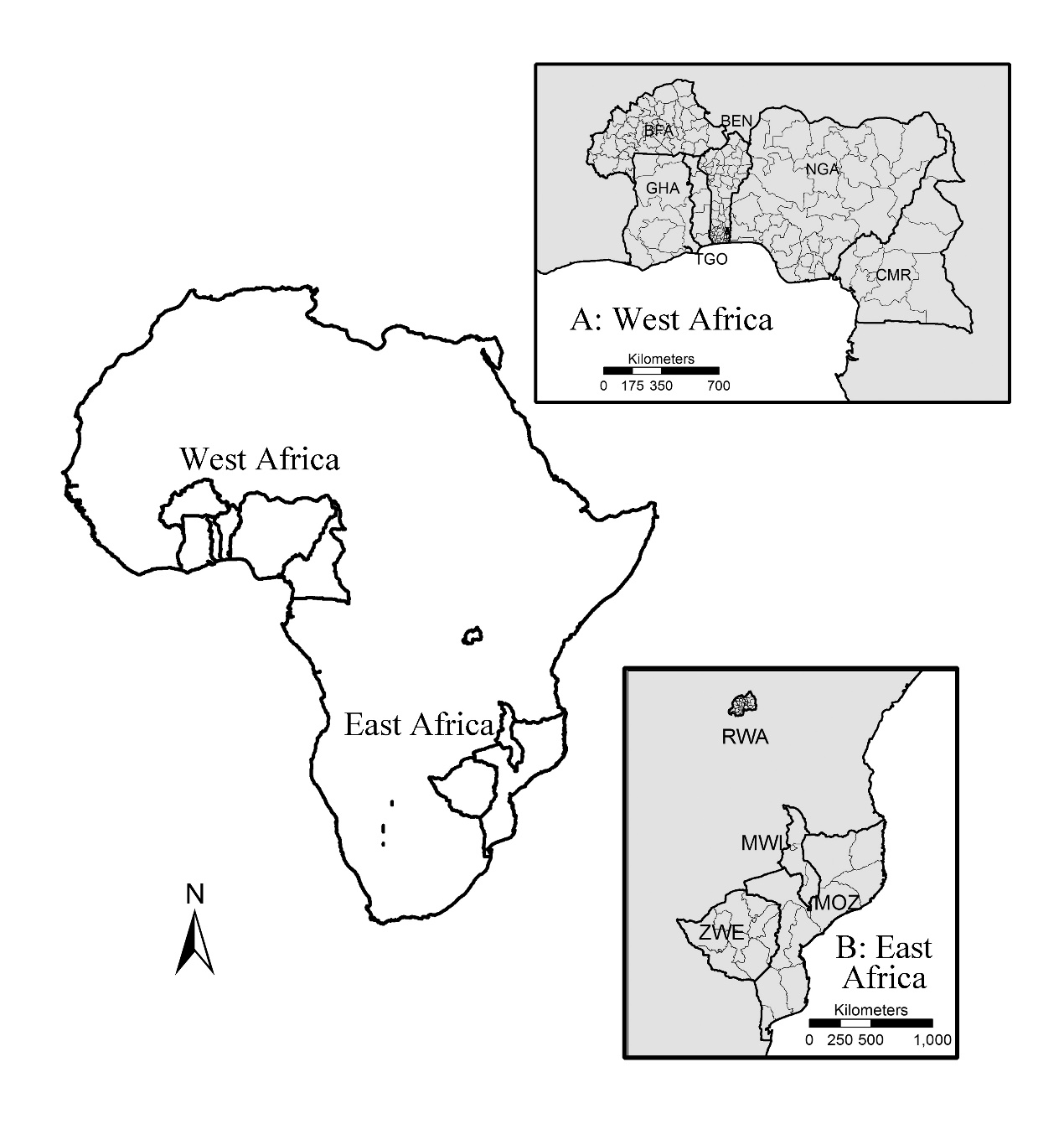

Supplement: Additional file 1: — Additional information for data used in analyses. (DOCX 242 kb) [file 13071_2016_1953_MOESM1_ESM.docx]
