# Supplementary material for: Regional level risk factors associated with the occurrence of African swine fever in West and East Africa
Source: Parasit Vectors. 2017 Jan 7;10:16. doi: 10.1186/s13071-016-1953-z (PMC5219763; doi:10.1186/s13071-016-1953-z)

# Supplementary Information (Additional File 2)

# Regional level risk factors associated with the occurrence of African swine fever in West and East Africa

Zheng Y.X. Huang^1,2*^, Frank van Langevelde^2^, Karanina J. Honer^2,3^, Marc Naguib^3^and Willem F. de Boer^2^

^1^College of Life Sciences, Nanjing Normal University, 210023 Nanjing, China

^2^Resource Ecology Group, Wageningen University, 6708PB Wageningen, the Netherlands

^3^Behavioural Ecology, Wageningen University, 6708WD Wageningen, the Netherlands

*Correspondence: zhengyxhuang@gmail.com

**Table S1:** Moran’s I values of residuals for the test of spatial autocorrelation in the best model both for West and East Africa.

| Year | The best model | | | |
| --- | --- | --- | --- | --- |
| Distance (k km) | global (0 – 2.5) | 0 – 0.5 | 0-1 | 0-1.5 |
| West Africa | 0.005** | 0.010* | 0.005** | 0.008** |
| East Africa | -0.010 | -0.007 | -0.001 | -0.009 |

* *P*< 0.05; ** *p* < 0.01; *** *p* < 0.001

**Figure S1:** Model predictions for the distribution of *Ornithodoros* *moubata* based on current climate conditions (A) and the ROC curve of the model (B).


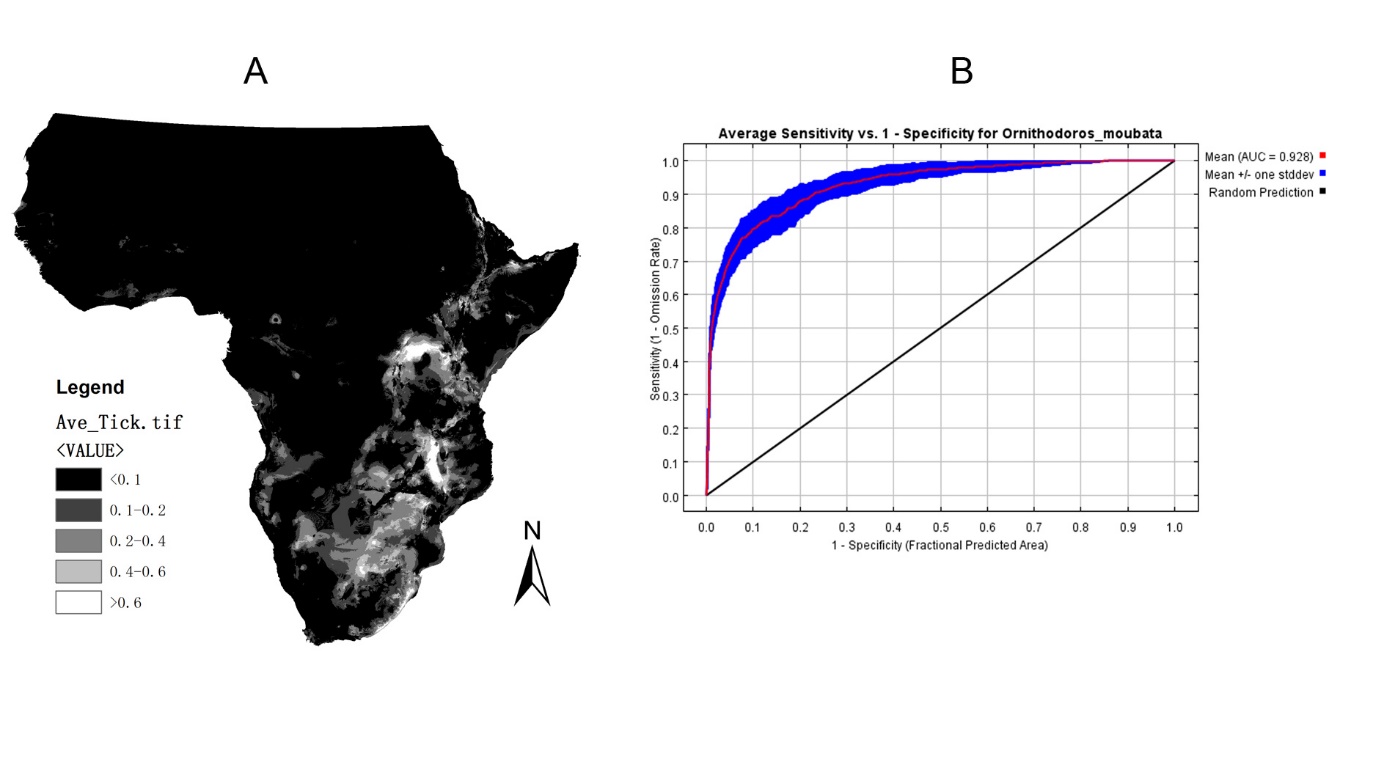

Supplement: Additional file 2: — Additional information for the results of analyses. (DOCX 154 kb) [file 13071_2016_1953_MOESM2_ESM.docx]
